# Improving face identification of mask-wearing individuals

**DOI:** 10.1186/s41235-022-00369-7

**Published:** 2022-03-28

**Authors:** Krista D. Manley, Jason C. K. Chan, Gary L. Wells

**Affiliations:** Iowa State University, Ames, USA

**Keywords:** Eyewitness identification, Lineups, Mask, Face covering, Face recognition

## Abstract

Research has consistently shown that concealing facial features can hinder subsequent identification. The widespread adoption of face masks due to the COVID-19 pandemic has highlighted the critical and urgent need to discover techniques to improve identification of people wearing face coverings. Despite years of research on face recognition and eyewitness identifications, there are currently no evidence-based recommendations for lineup construction for cases involving masked individuals. The purpose of this study was to examine identification accuracy of a masked perpetrator as a function of lineup type (i.e., unmasked or masked lineups) and perpetrator presence (i.e., absent or present). In both experiments, discriminability was superior for masked lineups, a result that was due almost exclusively to higher hits rates in target-present conditions. These data suggest that presenting a masked lineup can enhance identification of masked faces, and they have important implications for both eyewitness identification and everyday face recognition of people with face coverings.

## Introduction

The COVID-19 pandemic has made wearing a face mask commonplace around the world. This level of sudden and mass-scaled shift in human behavior is extremely rare in human history, and donning face coverings has profound implications for face recognition in everyday situations as well as eyewitness identification. Prior to 2020, wearing a face mask in public places and government buildings was sometimes unlawful (Noble, 2013) because it was often done by criminals of premeditated crimes to help conceal their identities. However, amid the worldwide pandemic, mask-wearing is required in many nations and might continue to some degree even after widespread distribution of vaccines (The White House, 2021). Consequently, identification of a masked perpetrator has become and will likely remain a major issue for the criminal justice system. Given the newfound scale of the problem, research on improving identifications for masked faces is urgently needed. Here, we examined whether presenting masked faces, as opposed to unmasked faces, *during retrieval* can improve identification of masked faces, whether this technique alters the association between witness confidence and identification accuracy, and whether witnesses prefer the type of lineup they receive.

Face identification based on memory (i.e., recognition) and perception (i.e., face matching) is impaired by mask-wearing (Carlson et al., 2021; Carragher & Hancock, 2020; Davies & Flin, 1984; Mansour et al., 2012) or disguises in general (Noyes & Jenkins, 2019; Shapiro & Penrod, 1986). Researchers have generally treated masking-wearing as an estimator variable (Cutler et al., 1987a, 1987b; Mansour et al., 2012; Shapiro & Penrod, 1986), because whether or not someone wears a mask when committing a crime is not under the control of the criminal justice system (Wells, 1978). Further, researchers have rarely considered how to mitigate the negative impact of masking on identification (Manley et al., 2019); rather, the purpose historically has been to measure the negative impact of masking on identification performance. Indeed, despite extensive science-based recommendations on the best ways to construct and conduct lineups (Wells et al., 2020), current lineup policies do not discuss recommendations for a masked perpetrator. Consequently, crime investigators must decide if or how to administer a lineup on a case-by-case basis.

An inspection of past cases reveals substantial variability in how investigators have handled lineup administrations for crimes that involved a masked perpetrator (Courteau, 1983; Egeler, 1977; Fierro, 1971; *Williams v State*, 1979). For example, in both *State v. Courteau* and *Williams v. State,* investigators administered an unmasked lineup to witnesses. However, in *Dupuie v. Egeler* (1977), after witnesses were unable to make an identification from an unmasked lineup, investigators asked the lineup members to put on a mask, after which the witnesses made identifications. Cases like these illustrate the importance of empirical evidence on the topic—because there are no evidence-based recommendations, investigators simply administer their preferred lineup based on intuition. Prior to 2020, the issue of face coverings had been of occasional concerns for the criminal justice system, but the COVID-19 pandemic and the subsequent widespread adoption of mask wearing has turned this problem into a global concern. Here, we argue that matching the lineup appearance to that of the perpetrator at the crime scene, even when the perpetrator was concealing their face with a mask, should improve eyewitness identification accuracy. We illustrate our theoretical rationale below.

### Transfer-appropriate processing and disguise

The principle of transfer-appropriate processing states that memory performance is best when the processes active during retrieval match those active during encoding (Morris et al., 1977; Tulving & Thomson, 1973). This idea has been realized in eyewitness memory studies, as reinstating the mental, environmental, or emotional context at retrieval that were present during encoding have generally benefited performance (Cutler et al., 1986, 1987b; Dalton, 1993; Gibling & Davies, 1988; Krafka & Penrod, 1985; Shapiro & Penrod, 1986; Toseeb et al., 2012). For example, reinstating the encoding context cues during retrieval improves performance both eyewitness identification and face recognition tasks (Shapiro & Penrod, 1986). Further, reinstating the encoding appearance of a target face in a lineup (e.g., beard) can improve identification performance (Davies & Flin, 1984; Foley & Foley, 1998; Hockley et al., 1999; Manley et al., 2019; Terry, 1994). Together, these studies suggest that the principles of transfer-appropriate processing might be applied to enhance eyewitness identification of a masked perpetrator. But what is transfer-appropriate processing in the context of faces?

A major theory of face perception posits that faces are processed in a holistic rather than a featural manner (Bartlett et al., 2003; Bruce & Young, 2012; Lampinen et al., 2012; Maurer et al., 2002; Tanaka & Farah, 1993; Tanaka & Simonyi, 2016). Both behavioral and neuroimaging evidence have provided substantial support for the holistic account of face processing (Harris & Aguirre, 2008; Kanwisher et al., 1997; Leder & Carbon, 2005; Tanaka & Farah, 1993; Tanaka & Sengco, 1997; Wilford & Wells, 2010; Yin, 1969; Yovel & Kanwisher, 2004). Of particular relevance here, some researchers have found that faces are automatically processed holistically (Hole, 1994; Young et al., 1987). When participants view facial “composites,” which consist of the top half of one face and the bottom half of a different face, they had more difficulty recognizing either half of the composite when the halves are aligned than when they are misaligned. The explanation is that when the halves are aligned, participants process the two halves as a single, holistic representation and are unable to ignore the other half of the composite.

The holistic account of face recognition provides theoretical foundation for why a masked lineup might increase identification accuracy of a masked perpetrator compared to an unmasked lineup. Several findings from the face recognition literature are particularly relevant to the current study. First, research using the part-whole paradigm provided evidence for holistic processing (Farah et al., 1995). Specifically, participants *encode a whole face* and then are asked to recognize individual features of that face (such as a nose), with these features presented as part of a face or as isolated parts. Participants were better at recognizing the old features when they appeared as part of a face than they were at recognizing the features in isolation. In contrast, when participants were asked to *encode features in isolation* and then recognized those features in a face or as isolated parts, they showed better recognition performance for a feature in isolation rather than as part of a whole face—an effect termed whole-face interference (Leder & Carbon, 2005). Leder and Carbon (2005) concluded that the optimal strategy to successfully recognize an individual feature would be to ignore the holistic representation of the face and rely on featural processing—a task that is likely very difficult when features are viewed in the context of a face (Young et al., 1987). Consequently, participants were unable to ignore the holistic representation when they perceived a whole face during retrieval, even though the existing memory trace included only a featural representation. When we consider the two aforementioned studies together, they form a classic 2 (encoding) × 2 (retrieval) transfer-appropriate pattern. That is, face recognition performance is improved when the processes at retrieval (i.e., featural or holistic) match those at encoding.

Applying this reasoning to the current research, when an eyewitness encounters a masked perpetrator, the face would be processed less holistically (Fitousi et al., 2021; Freud et al., 2020; McKone et al., 2006; Moscovitch et al., 1997).^1^ Therefore, administering an unmasked lineup would be considered transfer-*in*appropriate because the full-faces would elicit holistic processing. This processing mismatch can cause whole-face interference and decrease the witness’s ability to identify the masked perpetrator. Therefore, to recapitulate the processing orientation invoked during the encoding of the masked perpetrator, the lineup should also encourage more featural processing and less holistic processing. It is important to address the somewhat counterintuitive nature of this prediction. At the perceptual level, participants would see the same eyes regardless of whether they are shown a masked or an unmasked lineup. That is, the visible region in the masked face would be virtually identical to that of the unmasked face, but the unmasked face would provide *additional* information, including the bottom half of the nose, the lips, the jaw line, etc. Therefore, the transfer-appropriate processing account is making a *less-is-better* prediction, such that viewing less information during retrieval can improve identification performance of a masked person.

Using artificial faces, the present authors tested this idea in a picture recognition paradigm (Manley et al., 2019). Across four experiments, participants studied a computer-generated full face or a partial face that showed only the eyes. The latter was achieved either by cropping (in Experiment 1) or superimposing a static picture of a ski-mask on the face (in Experiments 2–4). Participants then attempted to identify the target from a recognition test that consisted of three full faces or three cropped/masked faces. The results revealed a transfer-appropriate pattern, such that identification performance was superior when the perceptual appearance of the lineup members matched the encoded target (e.g., encoded a partial face, retrieved from a partial-face lineup) compared to when it was mismatched (e.g., encoded a partial face, retrieved from a full-face lineup).

Although the Manley et al. (2019) finding hold promise for the idea that a masked lineup might improve identification of a masked face, the study had major weaknesses that severely limits the generalizability its results to eyewitness identification or real-life face recognition. First, Manley et al. used computer generated, contrived materials that were unrealistic in certain respects. All of the stimuli had identical hair, head shape, face shape, and skin tone, with very minor differences in the eyes, nose, and mouth. A second limit on generalizability is that Manley et al. used static pictures as their encoding stimuli, and the target pictures presented at encoding and in the target-present lineups were identical at the pixel level. This means that participants were performing a *picture recognition* task rather than a face recognition or eyewitness identification task. In the real world of eyewitness identification, however, the image used in a lineup is not identical to the encoded image of the culprit from the crime scene (which was a dynamically-moving image) even if the lineup contains the same culprit. Instead, the appearance of a face varies from moment to moment due to angles of view, lighting, distance, changes in facial expression, slight variations in hair style, and so on. The eyewitness identification task is a person recognition task, not a picture recognition task and within-person variabilities in appearance are critical aspects in the difficulties that can arise in person recognition (Burton, 2013; Jenkins et al., 2011; Russ et al., 2018). Given this limitation, the results from Manley et al. may only apply to picture memory and not eyewitness identification. To properly examine whether a masked lineup would yield superior eyewitness identification performance for a masked perpetrator, we extended Manley et al.’s design to a more ecologically valid paradigm with new materials (e.g. mock crime videos and real human faces instead of computer generated target and filler faces).

### Meta-memory and lineup preference

Because there are no recommended procedures for lineup administration in crimes involving a masked perpetrator, investigators might decide to ask the witness for their preference. It is therefore important to examine whether witnesses are aware of the putative advantage that a masked lineup might provide relative to an unmasked lineup, and if they would display a preference for one lineup type over the other. The issue is essentially one that deals with meta-memory—the ability for people to reason about their own memory (Dunlosky & Thiede, 2013).

In the context of eyewitness identification, there are two broad classifications of meta-memory judgments—prospective (pre-identification) and retrospective (post-identification) judgments (Chua et al., 2009; Dunlosky & Thiede, 2013; Fleming et al., 2016). The eyewitness literature is replete with studies examining retrospective judgments in the form of confidence ratings. The interest in retrospective confidence ratings makes sense given the serious implications of these ratings for identification accuracy and impact in the courtroom (Cutler et al., 1988, 1990; Wells et al., 1979). On the contrary, prospective judgments have received far less attention (Clark & Tunnicliff, 2001; Nguyen et al., 2018; Olsson & Juslin, 1999; Perfect, 2004; Saraiva et al., 2020; Sommer et al., 1995), even though these judgments can have a profound impact in the criminal justice system. For example, investigators often ask witnesses if they would recognize the perpetrator should they see the person again. If witnesses are overconfident in their own memory, then they might be more willing to make an identification even if the perpetrator is not in the lineup. The opposite could happen if witnesses are underconfident, because investigators might not administer a lineup at all. How eyewitnesses monitor their learning is uniquely important in eyewitness identification because witnesses cannot go back and re-study the to-be-retrieved information. In a meta-analysis of nine studies that included prospective confidence assessment, Cutler and Penrod (1989) concluded that prospective confidence judgments are weakly associated with identification accuracy and should not be used when determining whether to administer a lineup to a witness. We caution against over-relying on this assessment given that similar sentiment had been expressed about retrospective confidence judgment until relatively recently (Wells et al., 2020).

To assess lineup preference for the identification of a masked perpetrator and to further examine the association between confidence and accuracy, we asked participants to provide confidence before and after they were administered a lineup in Experiment 2. Do witnesses’ lineup preferences correspond to their meta-memory judgments (i.e., they should choose a masked lineup over an unmasked lineup if they perceive that they would have a better chance of identifying the perpetrator in the former), and are their prospective and retrospective confidence judgments diagnostic of actual performance?

### Experiment overview

Experiment 1 used a 2 (Lineup: masked lineup, unmasked lineup) × 2 (Target: absent, present) within-subjects design. All participants saw a masked perpetrator committing a crime and then attempted to identify that individual in either a masked or unmasked photo lineup. Note that we did not present participants with an unmasked perpetrator during the encoding phase and then varied lineup type at retrieval, because we deemed it extremely unlikely that a witness would be shown a masked lineup for an unmasked perpetrator. Manley et al. (2019), however, did include these conditions in their study, and they found that participants were better able to recognize the previously seen unmasked face in an unmasked lineup relative to a masked lineup.

**Fig. 1:**
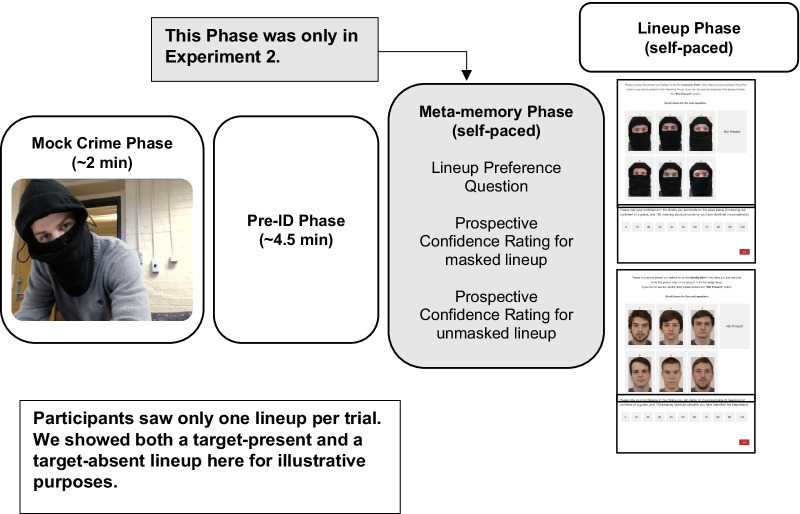
Depiction of a single trial. Each participant completed four trials, one for each of the four conditions

Participants completed one identification trial from each condition (masked target-present lineup, masked target-absent lineup, unmasked target-present lineup, unmasked target-absent lineup). The order of the trials was random, participants were not informed about which type of lineup they would see, and they were not told that they would complete four trials. In Experiment 1, each trial consisted of three phases (see Fig. 1).

First, participants watched a mock crime in which a perpetrator wore a mask that only revealed his eyes (Mock Crime Phase). Second, participants watched an unrelated, filler video in the Pre-ID Phase, which served as a brief retention interval. Third, participants were given a six-person lineup (masked or unmasked, target-absent or target-present) in the Lineup Phase. If participants chose the “Not Present” option, the same (but rearranged) lineup was re-presented and participants were asked to choose the person that looked the most like the perpetrator (to equate response criterion). After the Lineup Phase was complete, the next trial began with a new mock crime video. Experiment 2 employed the same design as Experiment 1, except that participants answered three prospective meta-memory questions before they viewed the lineup.

## Experiment 1

### Method

#### Participants

Participants were 314 undergraduate students from Iowa State University who participated in exchange for course credit. Table 1 contains more information about participant demographics.

**Table 1:** Participant demographics for Experiments 1 and 2

	Experiment 1	Experiment 2
Age		
Average	18.99 (1.36)	19.06 (1.89)
Range	18–29	18–43
Ethnicity		
American Indian or Alaska Native	< 1%	< 1%
Black or African American	5%	3%
Chose not to respond	< 1%	< 1%
East Asian	2%	2%
Hispanic or Latino/a	6%	4%
Other	2%	2%
South/Southeast Asian	3%	2%
West Asian/middle eastern	0%	< 1%
White/Caucasian	80%	84%
Gender		
Man	38%	37%
Woman	60%	62%
Other	< 1%	< 1%
Chose not to respond	< 1%	< 1%

Values in parentheses represent standard deviations

Four participants were excluded from analyses due to video playback problems. Therefore, 310 participants were included in final data analyses. Data from 214 participants were collected in the lab and data from 96 participants were collected online. All participants, regardless of whether they completed the experiments online or in-lab, were Iowa State University undergraduate students. Participation location (online, in-lab) did not affect our results for either experiment (all *F*s < 1.21, *p*s > 0.271), so we collapsed across this variable when reporting our data. We determined the sample size based on an exact proportions McNemar analysis which used the most conservative odds ratios and discordant pairs from Manley et al., (2019; Experiments 3 and 4). Specifically, the predicted necessary sample size (*n* = 282) was computed using G*Power (Faul et al., 2007) with power set at 0.85, a two-tailed value of *α* = 0.05, discordant pairs = 0.53, and an OR = 1.65.

**Fig. 2:**
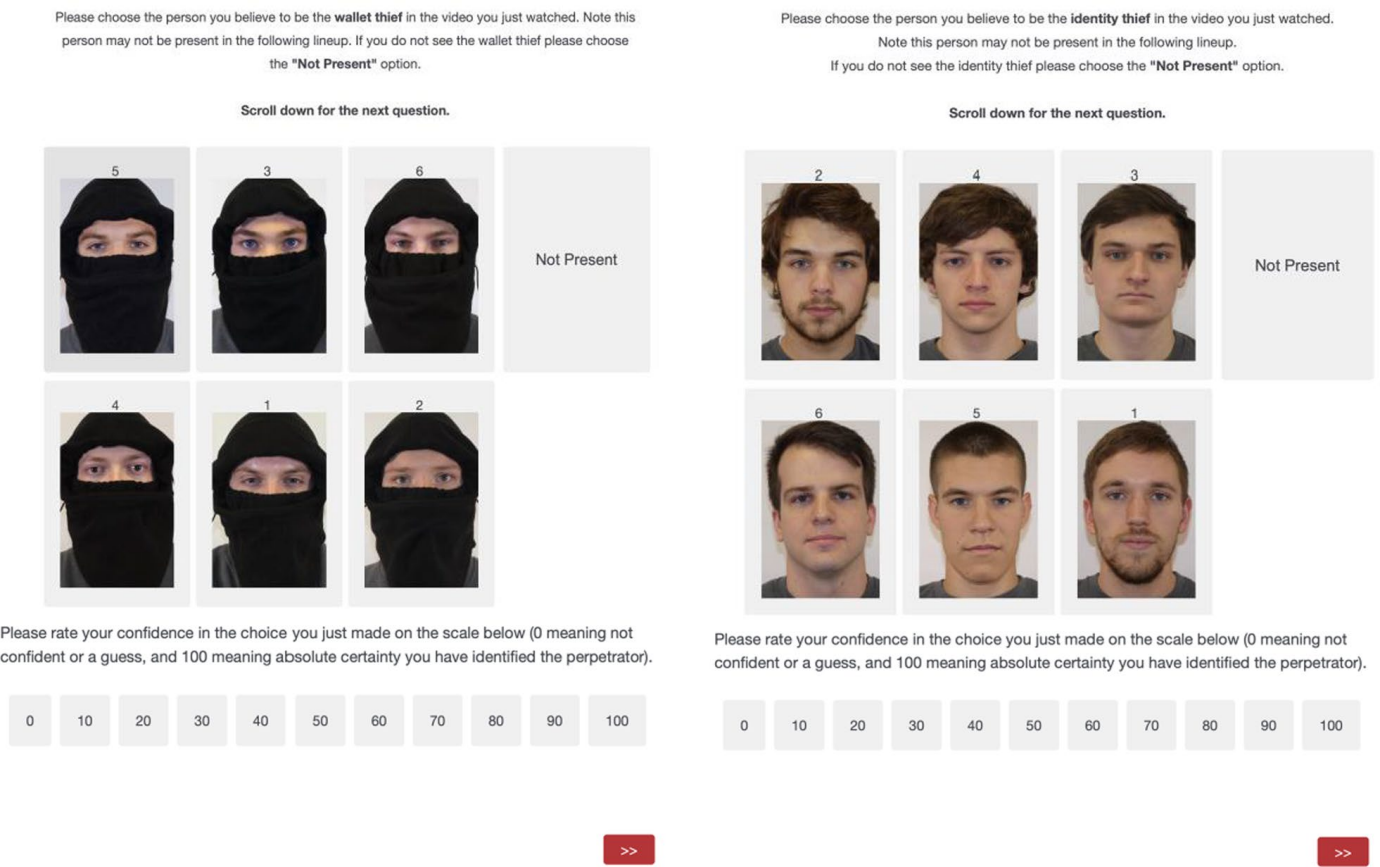
An example of a masked lineup and an unmasked lineup. The two lineups shown here involved different perpetrators

Materials and Procedure. We created four mock crime videos for this study. Each video depicted a different theft. The four videos showed a man stealing a credit card and identity information from a victim, a wallet theft from an unlocked car, a computer theft from an empty office, and bike theft on a college campus, respectively. Each crime featured a different, college-age, male Caucasian perpetrator wearing a black mask that revealed only the area around his eyes (see Figs. 1 and 2). We opted to use a mask that covered all outer and lower facial features. We believe that this type of occlusion would produce the strongest effect because it would have the largest influence on holistic processing. Further, the same mask was used in every mock crime as well as all lineup photos to ensure participants would not use differences in the mask for their identification decision. Each video lasted approximately two minutes (*M* = 122 s), and the masked perpetrator was on screen for approximately 75 s, with a close-up view that lasted a total of about half a minute (*M* = 25 s). Assignment of the videos to lineup conditions was counterbalanced across participants.

Participants began by watching a mock crime video and were told to attend to the perpetrator of the videos. After each mock crime video, participants watched a distractor video and answered two questions intended as an attention check. The first asked “what was the overall message of the video?” and the second asked “what was one lesson that the main character learned by the end?” Each of the four ~ 4.5 min dialogue-free distractor videos were randomly assigned to each trial. After each distractor video, participants were presented with a six-person lineup (see Fig. 2).

The target-present lineup included five filler faces and the target-absent lineup replaced the perpetrator with an additional filler. The position of the target and each filler face was randomized every trial and for each participant. The filler faces were selected for each perpetrator via pilot testing with 156 participants from Amazon Mechanical Turk. For the pilot, participants watched each of the four videos and immediately attempted to choose the target from either an unmasked (*n* = 81) or masked (*n* = 75), target-absent eight-person lineup. The final lineup members were selected by removing the faces with the highest and lowest selection rates from both lineup types. We chose to implement the above method in lieu of a simpler method in which fillers were chosen based on a verbal description because of the challenge in creating such a description for a masked face (e.g., “he wore a mask and had blue eyes”).

In the present experiment, participants were told that the perpetrator might not be present, and in this case, they should choose “Not Present.” After making their selection, participants rated their (post-ID) confidence on a 11-interval scale from 0 to 100, with 0 indicating not sure at all or a guess and 100 indicating absolute certainty. If participants selected the “Not Present” option, they received the same lineup again but with a fresh random order for the faces, and they were told to “choose the individual who most closely resembles the thief.” We included this additional trial to simulate a biased lineup procedure (i.e., when witnesses are discouraged from choosing no one), and the results from this procedure resembled those from the initial trial (i.e., unbiased lineup). Therefore, we focused on the initial identification trial, but the forced-choice lineup data are available on the Open Science Framework (OSF) at https://osf.io/5f68j/?view_only=912a09bd44954de58c41336a7b74fa33. After participants completed all four trials, they were given a short demographics questionnaire were asked if they knew any of the actors or lineup members, debriefed, and dismissed. None of the participants indicated that they recognized any of the actors in the mock crime videos or the lineups in Experiment 1.

### Results

Because a target-present and target-absent lineup require a fundamentally different correct response (target identification vs. correct rejection), we analyzed the data from these lineups separately using Cochran’s *Q* (an extension of McNemar *χ*^2^ for related samples; Field, 2009). Data are reported starting with target-present lineups followed by target-absent lineups. We then examined the data using Confidence-Accuracy Characteristic curves. Finally, we conducted a compound signal detection model (SDT-CD) analysis of the data. The stimuli and data for each experiment can be found on the aforementioned OSF page.

### Target-preset lineup

The most important finding is that participants were more likely to identify the perpetrator from a masked lineup (*M* = 0.58) compared to an unmasked lineup (*M* = 0.48), *χ*^2^(310) = 6.72, *p* = 0.010, OR = 0.63, CI [0.44, 0.91] (see top of Table 2). They were also less likely to pick a filler from a masked lineup (*M* = 0.28) than an unmasked lineup (*M* = 0.36), *χ*^2^(310) = 6.15, *p* = 0.013, OR = 1.62, CI [1.09, 2.44]. Lastly, lineup type did not affect incorrect rejections (*M*_masked_ = 0.14, *M*_unmasked_ = 0.15), *χ*^2^ < 1, *p* = 0.637.

**Table 2:** Identification proportions, confidence ratings, discriminability and response bias for identification decisions in Experiment 1

	Masked Lineup	Unmasked Lineup
*Target-present lineups*		
Identification proportions		
Target IDs	.58 (.49)*	.48 (.50)
Filler IDs	.28 (.45)*	.36 (.48)
Incorrect rejections	.14 (.35)	.15 (.36)
Confidence ratings		
Target IDs	74% (21)*	69% (23)
Filler IDs	63% (19)*	56% (23)
Incorrect rejections	65% (27)	62% (24)
*Target-absent lineups*		
Identification proportions		
Correct rejections	.25 (.43)	.31 (.46)
Filler IDs	.75 (.43)	.69 (.46)
Confidence ratings		
Correct rejections	64% (25%)	64% (25%)
Filler IDs	63% (21%)*	57% (22%)
Discriminability and Bias		
*d′*	1.55 (.09)*	1.32 (.07)
*c*	− 0.59 (− .03)	− 0.49 (− .03)

Values in parentheses are standard deviations except in the cases of *d’* and *c*, for which the parenthetical values are standard errors. An asterisk indicates a significant difference between lineup types

Linear Mixed Models (LMM) ANOVAs were used to examine the effects of lineup type on confidence ratings (see bottom of Table 2). Participants were more confident about their correct identification in a masked lineup (*M*_masked_ = 74%) than in an unmasked lineup (*M*_unmasked_ = 69%), *F*(1, 326) = 4.69, *p* = 0.031, *d* = 0.23. But the same pattern also occurred for filler identifications (*M*_masked_ = 63% vs. *M*_unmasked_ = 56%), *F*(1, 198) = 4.31, *p* = 0.039, *d* = 0.28. However, confidence ratings for incorrect rejections from masked lineups (*M* = 65%) and unmasked lineups (*M* = 62%) were comparable, *F*(1, 90) = 0.30, *p* = 0.585, *d* = 0.10. Therefore, the masked lineup appeared to have increased confidence when participants chose someone, regardless of whether that person was the perpetrator.

### Target-absent lineup

Participants correctly rejected target-absent unmasked lineups (*M* = 0.31) at a slightly higher rate than masked lineups (*M* = 0.25, see Fig. 4). However, this effect did not reach significance (and was not found in Experiment 2), *χ*^2^(310) = 3.14, *p* = 0.076, OR = 1.40, CI [0.95, 2.10]. In a target-absent lineup, filler identifications are complementary to correct rejections (e.g., correct rejections + filler identifications = 1), so they were not analyzed separately.

Participants provided virtually identical confidence ratings for correct rejections made from a masked lineup (*M* = 64%) and an unmasked lineup (*M* = 64%), *F* < 1, *p* = 0.906, *d* = 0.02, but they were more confident in their filler identifications made from masked lineups (*M* = 63%) than unmasked lineups (*M* = 57%), *F*(1, 445) = 9.28, *p* = 0.002, *d* = 0.25. Once again, these data suggest that the masked lineup increased confidence when participants choose someone from a lineup regardless of accuracy.

### Confidence-accuracy characteristic (CAC)

CAC curves (Wixted & Wells, 2017) provide a visualization of suspect (both guilty and innocent) identification accuracy at each level of confidence:

$$Suspect ID accuracy =100\% \times n{SID}_{TP-C}/(n{SID}_{TP-C}+(n{FID}_{TA-C}/n)$$

*nSID*_*TP-c*_ is the number of target identifications made from a target-present lineup (i.e., guilty-suspect IDs) at each level of confidence *c*. Because there were too few observations at each confidence levels, we binned the confidence levels from 11 to four so that each contained a roughly equal number of responses. The four confidence levels corresponded to 0–40, 50–60, 70–80, 90–100. The term *nFID*_*TA-c*_ is the number of filler identifications made from target-absent lineups (i.e., IDs of non-targets) at the same level of confidence. Because there was no designated innocent suspect in the current study, *nFID*_*TA-c*_ is divided by the lineup size in the target-absent lineup (*n* = 6 in the current study) to estimate the innocent suspect frequencies. Suspect identification accuracy is then plotted at each level of confidence to show a CAC curve for each condition. The CAC curve offers a visual inspection of the relationship between accuracy and confidence (i.e., do higher eyewitness confidence ratings indicate greater identification accuracy?), and we are particularly interested in whether masked and unmasked lineups affect this relationship.

**Fig. 3:**
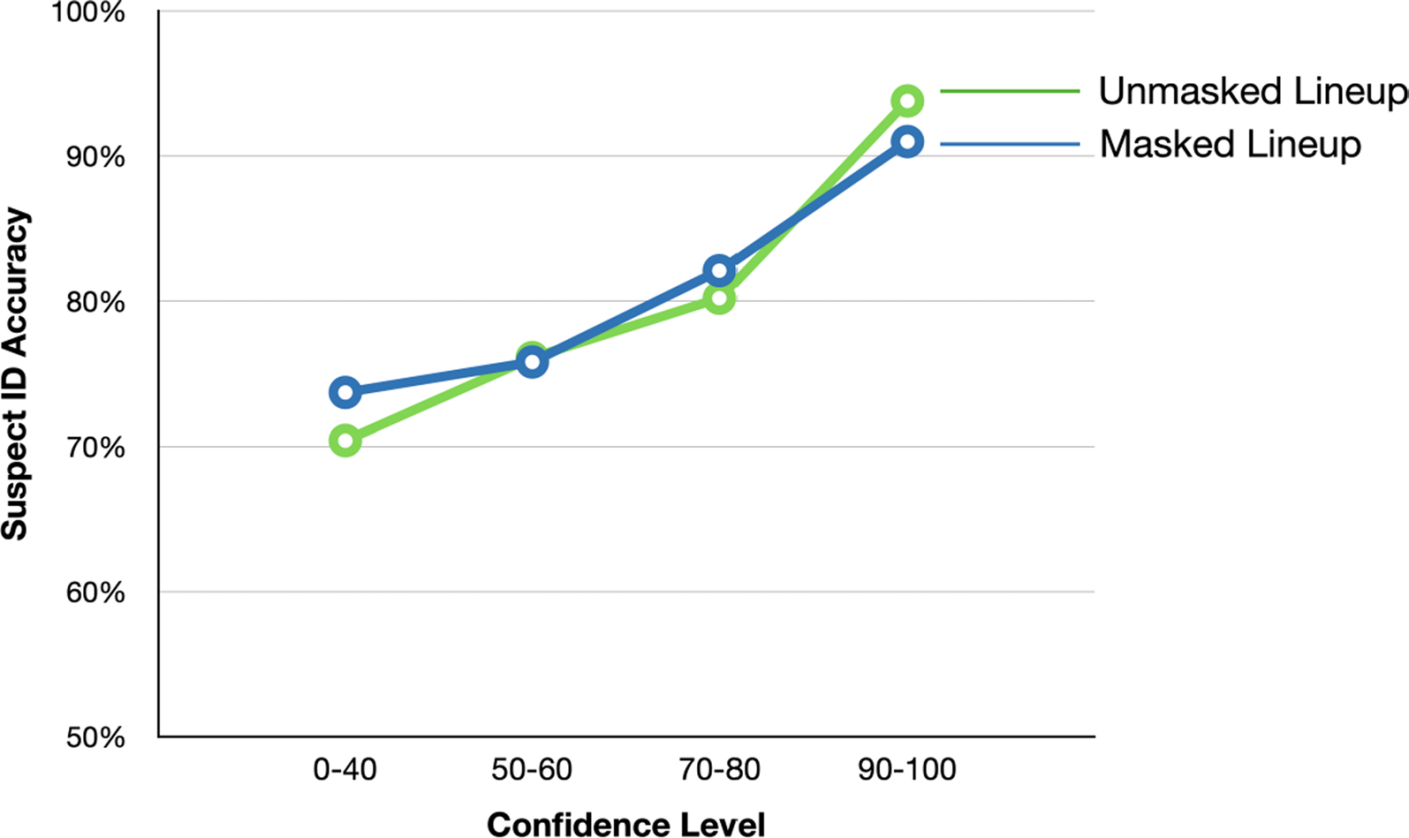
Confidence-Accuracy Characteristics (CAC) curves for the masked and unmasked lineups in Experiment 1. The y-axis starts at 50%. Note that the values that contribute to a CAC curve are proportions from the whole sample and so CAC curves do not have error bars

As shown in Fig. 3, the CAC curves have a positive slope, such that suspect identification accuracy rose across confidence levels, but the more important finding is that the masked-face lineup and full-face lineup produced overlapping CAC curves – i.e., the masked lineup increased target identifications relative to the unmasked lineup, and the confidence judgments from the two lineups provided similar diagnostic value.

In general terms, the data in Fig. 3 tend to corroborate the claim (Wixted & Wells, 2017) that high levels of eyewitness identification confidence on suspect identifications (e.g., > 90%) tend to be associated with high levels of accuracy (also > 90%) even when overall accuracy is impaired.

### Compound signal detection model

A compound signal detection model (SDT-CD) examined whether lineup type affected discriminability (Palmer & Brewer, 2012; Palmer et al., 2010). The model consists of both detection (i.e. choosing *someone* in target-present lineups and rejecting target-absent lineups) and identification (i.e. choosing the target). The model produces a discriminability (*d’*) and response bias (*c*) value for each condition. Negative (*c*) values indicate a conservative bias and positive values indicate a liberal bias. We adopted the integration decision rule, which offers a good fit for eyewitness identification data (Palmer & Brewer, 2012). To make inferences, *G*^2^ (similar to *χ*^2^) for the model where *d'* (or *c*) was constrained is subtracted from the *G*^2^ for the model where *d'* was permitted to differ. For example, if the *G*^*2*^ values were equal to 10 for the fixed model and 5 for the model in which *d’* was allowed to vary, the difference of 5 would exceed the critical value for *χ*^2^(1) = 3.84, and we can infer that restricting the two lineup conditions to a single *d'* value significantly impaired model fit and it is unlikely that the two lineup conditions have the same *d'* value.

Results for *d'* and *c* are displayed in Table 2, and they showed that discriminability was higher when participants were administered a masked lineup (*d'* = 1.55) compared to an unmasked lineup (*d'* = 1.32), *G*^*2*^(1) = 6.05, *p* = 0.014. Response bias, in contrast, did not differ significant across the lineups, and both showed conservative leaning, negative values (*c*_masked_ = -0.59, *c*_unmasked_ = − 0.49), *G*^*2*^(1) = 1.68, *p* = 0.195.

### Discussion

Several important results emerged from Experiment 1. First, presenting participant witnesses with a masked lineup improved their ability to identify the target when the perpetrator was present (relative to an unmasked lineup), but it did not affect correct rejections when the perpetrator was absent. Second, identifications made from a masked lineup were associated with greater confidence than those made from an unmasked lineup, regardless of whether those identifications were correct (i.e., target ID) or not (i.e., filler ID), but lineup type did not affect confidence for rejections. Third, both the masked and unmasked lineup produced overlapping, positive CAC curves, such that lineup types did not affect the diagnosticity of confidence.

In the Introduction, we noted that Manley et al. (2019) showed that administering a masked lineup can improve eyewitness identification of a masked perpetrator relative to the default, unmasked lineup, but the Manley et al. study used a picture recognition task, in which the lineup images were pixel-perfect reproduction of the images seen during encoding. In the present study, we used more ecologically valid materials and also found that a masked lineup increased target identifications from a perpetrator-present lineup. However, there were also some notable differences between our results and those reported by Manley et al. For example, whereas Manley et al. found that a masked lineup increased correct rejections relative to an unmasked lineup when the target was absent (see their Experiment 3), we found no such difference in our experiment. Moreover, we found that a masked lineup increased confidence for both target- and filler-IDs relative to an unmasked lineup, but Manley et al. found the opposite for filler-IDs made from a target-present lineup. More research is needed to ascertain the reasons for these discrepancies. For present purposes, however, the most important finding is that administration of a masked lineup increased target identifications and overall discriminability without influencing the diagnostic value of confidence ratings.

## Experiment 2

Experiment 2 has two purposes. First, we wanted to replicate the findings from Experiment 1. Second, we wanted to examine whether participant witnesses had a preference for lineup types, and if not, whether they would develop such a preference once they have experienced the eyewitness identification task. We also examined whether prospective confidence judgments (similar to judgments of learning) can predict identification accuracy, given the positive diagnosticity of retrospective confidence judgments. Immediately before the presentation of each lineup in Experiment 2, participants were first asked to indicate their lineup preference (i.e., masked lineup or unmasked lineup); they were then asked to make prospective confidence judgments for *both lineup types*.

## Method

### Participants and Design

Participants were 334 Iowa State University students. Data from 21 of the 334 participants were not included in the analyses because they failed attention checks (*n* = 13), because they indicated that they knew one of the actors or fillers (*n* = 5), or because total duration spent completing the study was over twice the maximum amount of time (*M* = 45 min, *Max* = 58 min) it took in-lab participants (*n* = 2). Of the remaining 313 participants, 158 completed the study in the lab and 155 completed the study online through the university SONA system. For more information about participant demographics, see Table 1.

### Materials and procedure

All materials and procedures were identical to Experiment 1 except that participants were asked three questions immediately before each identification task. The first was the lineup preference question, “You are going to see a lineup. The lineup will either consist of unmasked faces or faces wearing ski masks that expose the eyes. The computer randomly determines which version you will see. But if you were given a choice, would you like to see a lineup of unmasked faces or a lineup of faces wearing masks?” Participants could choose “a lineup of unmasked faces” or “a lineup of faces wearing masks.” Next, two prospective confidence questions were asked in a random order. The two questions differed by only the italicized words as shown here: “If you were asked to identify the perpetrator in a 6-person lineup consisting of *faces wearing masks/unmasked faces*, that may or may not contain the perpetrator, how likely do you think you would be able to make a correct decision? Please rate your answer on a scale from 0–100, 0 indicating no chance of correct decision, 100 indicating absolute certainty of correct decision?”.

## Results and discussion

### Target-preset lineup

The most important finding was that participants identified the perpetrator more often from the masked lineup (*M* = 0.53) compared to the unmasked lineup (*M* = 0.38), *χ*^2^(313) = 14.30, *p* < 0.001, OR = 0.53, CI [0.37, 0.75] (see top of Table 3). Moreover, participants made significantly fewer filler identifications from the masked lineup (*M* = 0.31) than from the unmasked lineups (*M* = 0.41), *χ*^2^(313) = 8.53, *p* = 0.003, OR = 1.73, CI [1.18, 2.56], and there was no significant difference for incorrect rejections across lineup types (*M*_masked_ = 0.16) compared to unmasked lineups (*M*_unmasked_ = 0.21), *χ*^2^(313) = 2.04, *p* = 0.153. These results replicated those from Experiment 1, except that they favored the masked lineup over the unmasked lineup even more.

**Table 3:** Identification proportions, confidence ratings, discriminability, and response bias for identification decisions in Experiment 2

	Masked lineup	Unmasked lineup
*Target-present lineups*		
Identification proportions		
Target IDs	.53 (.50)*	.38 (.49)
Filler IDs	.31 (.46)*	.41 (.49)
Incorrect rejections	.16 (.37)	.21 (.41)
*Confidence ratings*		
Target IDs	68% (24%)	64% (22%)
Filler IDs	55% (23%)*	49% (22%)
Incorrect rejections	56% (21%)	49% (23%)
*Target-absent lineups*		
Identification proportions		
Correct rejections	.38 (.49)	.40 (.49)
Filler IDs	.62 (.49)	.60 (.49)
Confidence ratings		
Correct rejections	59% (19%)	57% (23%)
Filler IDs	58% (21%)*	53% (22%)
Discriminability and Bias		
*d*′	1.54 (.09)*	1.13 (.06)
*c*	− 0.33 (− .02)	− 0.30 (− .02)

Values in parentheses are standard deviations except in the cases of *d'* and *c*, for which the parenthetical values are standard errors. An asterisk indicates a significant difference between lineup types

Linear mixed models ANOVA were performed to examine differences in retrospective confidence ratings for target identifications, filler identifications, and incorrect rejections as a function of lineup type (see bottom of Table 3). Participants gave quantitatively, but not significantly, higher confidence ratings for target-IDs made from a masked lineup (*M* = 68%) than from an unmasked lineup (*M* = 64%), *F*(1, 282) = 2.88, *p* = 0.091, *d* = 0.20. They also rated their filler-IDs from a masked lineup with greater confidence (*M* = 55%) than those from an unmasked lineup (*M* = 49%), *F*(1, 224) = 4.15, *p* = 0.043, *d* = 0.27. There was no significant difference in confidence ratings for incorrect rejections made from a masked (*M* = 56%) compared to an unmasked lineup (*M* = 49%), *F*(1, 114) = 2.52, *p* = 0.115, *d* = 0.30. These data largely replicated those from Experiment 1.

### Target-absent lineup

When the perpetrator was absent, participants were similarly likely to reject a masked lineup (*M* = 0.38, SD = 0.49) as they were an unmasked lineup (*M* = 0.40, SD = 0.49), *χ*^2^(313) = 0.30, *p* = 0.587, OR = 1.10, CI [0.76–1.60]. Once again, we did not analyze the data for filler identifications separately because they are the mathematical complement of correct rejections in a target-absent lineup.

Retrospective confidence ratings for correct rejections did not differ across lineup types (*M*_masked_ = 59%, *M*_unmasked_ = 57%), *F* < 1, *p* = 0.556, *d* = 0.07, but filler identifications made in a masked lineup were associated with greater confidence (*M* = 58%) than those made in an unmasked lineup (*M* = 53%), *F*(1, 382) = 4.38, *p* = 0.037, *d* = 0.22. These data once again showed that the masked lineup increased confidence for identifications but not for rejections.

### Confidence-accuracy characteristic (CAC)

**Fig. 4:**
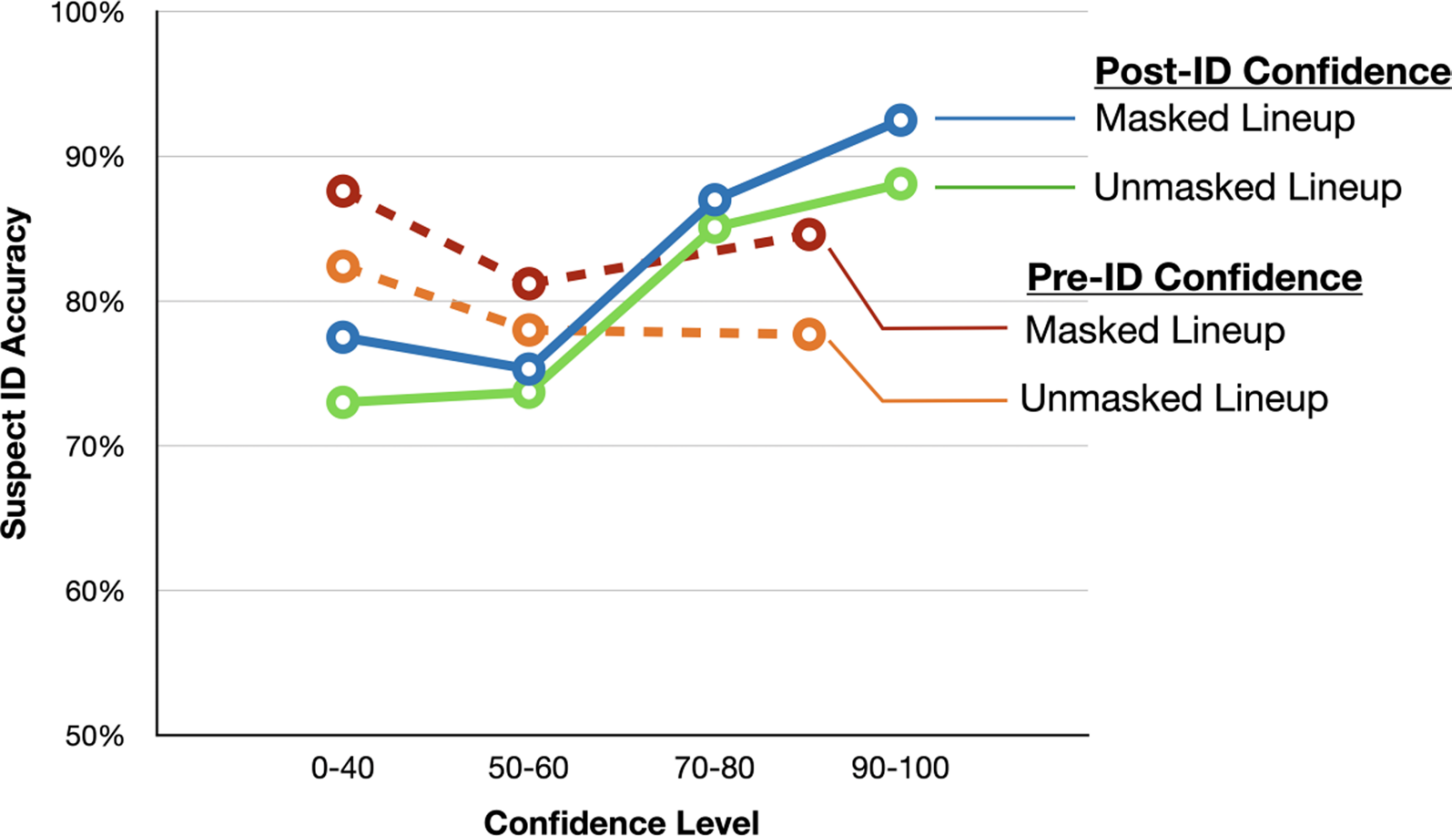
Confidence-Accuracy Characteristics (CAC) curves for the masked and unmasked lineups in Experiment 2. The solid curves are based on post-ID confidence (like those in Experiment 1) and the dotted curves are based on pre-ID confidence (applicable to only Experiment 2). The pre-ID confidence data were binned into confidence levels from 0–40, 50–60, and 70–100. The y-axis starts at 50%. Note that the values that contribute to a CAC curve are proportions from the whole sample and so CAC curves do not have error bars

Figure 4 displays the retrospective CAC curves. Similar to Experiment 1, the positive slopes show that higher confidence was indicative of greater suspect identification accuracy.

More importantly, masked- and unmasked lineups produced similar CAC curves, such that the beneficial effects of the masked lineup over the unmasked lineup on target identifications did not appear to come at the expense of poorer diagnosticity of the confidence judgments.

### Compound signal detection model

The masked lineup led to greater discriminability (*d’* = 1.54, SE = 0.09) than the unmasked lineup (*d’* = 1.13, SE = 0.06; *G*^*2*^(1) = 18.05, *p* < 0.01; see Table 3). Further, response bias was similar and conservative-leaning for both lineup types (*c*_masked_ = − 0.33, SE = − 0.02, *c*_unmasked_ = − 0.30, SE = 0.02; *G*^*2*^(1) = 0.26, *p* = 0.611).

### Lineup preference and prospective confidence judgments

We collapsed across the variable of perpetrator presence (i.e., target-present vs. target-absent lineup) when analyzing lineup preference and prospective confidence, because these measures were taken before participants saw the lineup (see Table 4).

**Table 4:** Lineup preference proportions and prospective confidence ratings across trials in Experiment 2

	Trial 1	Trial 2	Trial 3	Trial 4
Lineup preference (%)				
Masked lineups	53	72	70	72
Unmasked lineups	47	28	30	28
Trial 1: Masked lineup				
Masked lineups	53	71	70	68
Unmasked lineups	47	29	30	32
Trial 1: unmasked lineup				
Masked lineups	53	72	70	76
Unmasked lineups	47	28	30	25
Prospective confidence (%)				
Masked lineups	53 (22)	56 (20)	54 (21)	52 (20)
Unmasked lineups	53 (23)	51 (21)	48 (20)	47 (21)

Values in parentheses represent standard deviations

On the first trial, participants displayed no significant preference, such that the masked lineup (*M* = 53%) and unmasked lineup (*M* = 47%) were preferred by roughly half of the participants, respectively, *χ*^2^(313) = 1.41, *p* = 0.235. However, from the second trial onward, participants developed a preference for the masked lineup (*M* = 72%), *χ*^2^(313) = 58.23, *p* < 0.001, which persisted for Trial 3 (70%), *χ*^2^(313) = 51.53, *p* < 0.001, and Trial 4 (72%), *χ*^2^(313) = 59.97, *p* < 0.001. Intriguingly, this pattern occurred regardless of whether participants received an unmasked or masked lineup during their first trial (see Table 4).

The prospective confidence ratings also demonstrated the same preference development. On Trial 1, participants rated their likelihood of a correct decision as virtually identical between a masked lineup and an unmasked lineup (both *M*s = 53%), *t*(312) = 0.56, *p* = 0.567, *d* = 0.04. However, by Trial 2, participants began reporting higher prospective confidence for the masked lineup (*M* = 56%) than the unmasked lineup (*M* = 51%), *t*(312) = 4.23, *p* < 0.001, *d* = 0.24, and this difference was maintained on Trial 3 (54% vs. 48%), *t*(312) = 5.61, *p* < 0.001, *d* = 0.32, and Trial 4 (52% vs. 47%), *t*(312) = 4.55, *p* < 0.001, *d* = 0.26.

We conducted an additional 2 (Lineup: masked, unmasked) × 2 (Lineup Choice: chosen, not chosen) LMM ANOVA to assess the relationship between lineup preference and prospective confidence. If participants’ preference for lineup type was driven by, or associated with, the perceived efficacy of the lineup, then they should report a higher prospective confidence for their preferred lineup over their non-preferred lineup. This was indeed the case, *F*(1, 1248) = 39.92, *p* < 0.001, such that participants provided higher prospective confidence ratings for the lineup that they chose (*M* = 55%) relative to the one they did not choose (*M* = 48%). There was not a significant effect of lineup or an interaction, *F*s < 1, *p*s > 0.534. Importantly, whether participants were presented with the lineup they chose or did not choose did not impact accuracy as performance patterns were similar to those presented above for all four lineup types (see Table 5 for means, *F*s < 1.68, *p*s > 0.195).

**Table 5:** Identification proportions as a function of lineup type and preferences in Experiment 2

	Preferred	Not preferred
*Target-present lineups*		
Masked		
Target IDs	.54 (.50)	.51 (.50)
Filler IDs	.31 (.47)	.30 (.46)
Incorrect rejections	.15 (.36)	.19 (.40)
Unmasked		
Target IDs	.37 (.49)	.39 (.50)
Filler IDs	.45 (.50)	.39 (.49)
Incorrect rejections	.18 (.39)	.22 (.42)
*Target-absent lineups*		
Masked		
Correct rejections	.39 (.49)	.36 (.48)
Filler IDs	.61 (.49)	.64 (.48)
Unmasked		
Correct rejections	.33 (.47)	.43 (.50)
Filler IDs	.67 (.47)	.57 (.50)

Values in parentheses represent standard deviations

Finally, CAC curves for prospective confidence were plotted using dotted lines in Fig. 4. We binned the prospective CAC curves into three intervals (rather than four intervals, as was the case for the retrospective CAC curves) to ensure that we have enough, and roughly equal number of data points in each interval. A striking contrast between the diagnosticity of prospective and retrospective confidence emerged. Whereas the retrospective CAC curves showed that higher confidence was indicative of greater suspect identification accuracy, the prospective CAC curves showed no much pattern. The flat CAC curves here indicate that prospective confidence was not predictive of subsequent identification accuracy, and this conclusion applied to both the masked and unmasked lineups.

### Combined data: by-item actor analysis

To assess if the specific actors impacted the pattern of results, we combined the data from Experiments 1 and 2. This was to ensure adequate power as the data will be split four ways, decreasing reliability, and increasing variability.

For target-present lineups, actor interacted with lineup type, *F*(1, 1238) = 4.89, *p* = 0.002. Specifically, for the computer thief (*M*_MF_ = 0.67, SD_MF_ = 0.47; *M*_FF_ = 0.46, SD_FF_ = 0.50), the identity thief (*M*_MF_ = 0.50, SD_MF_ = 0.50; *M*_FF_ = 0.37, SD_FF_ = 0.49), and the wallet thief (*M*_MF_ = 0.47, SD_MF_ = 0.50; *M*_FF_ = 0.27, SD_FF_ = 0.44), participants made more target identifications for masked-face lineups (*M* = 0.55, SD = 0.50) compared to full-face lineups (*M* = 0.43, SD = 0.50); however, for the bike thief (*M*_MF_ = 0.57, SD_MF_ = 0.50; *M*_FF_ = 0.63, SD_FF_ = 0.49), participants made fewer target identifications from masked-face lineups compared to full-face lineups. This last difference was further examined through a post-hoc comparison which found that the difference in target identifications was not significant, *F*(1, 308) = 0.86, *p* = 0.355.

For target-absent lineups, actor interacted with lineup type, *F*(1, 1238) = 4.11, *p* = 0.007. Specifically, for the bike thief (*M*_MF_ = 0.35, SD_MF_ = 0.48; *M*_FF_ = 0.50, SD_FF_ = 0.50), the identity thief (*M*_MF_ = 0.24, SD_MF_ = 0.43; *M*_FF_ = 0.30, SD_FF_ = 0.46), and the wallet thief (*M*_MF_ = 0.20, SD_MF_ = 0.40; *M*_FF_ = 0.26, SD_FF_ = 0.44), participants made more correct rejections from full-face lineups (*M* = 0.31, SD = 0.46) compared to masked-face lineups (*M* = 0.35, SD = 0.48); however, for the computer thief (*M*_MF_ = 0.46, SD_MF_ = 0.50; *M*_FF_ = 0.36, SD_FF_ = 0.48), participants made more correct rejections from masked-face lineups compared to full-face lineups.

## Experiment 2 discussion

The purpose of Experiment 2 was to replicate the data from Experiment 1 and to gain insight into participant’s beliefs about their own memory for a masked perpetrator. Would participants have a preference for a masked lineup or for an unmasked lineup, and would their preference change with experience? Our results showed that participants did not have a preconceived notion of the superiority of the masked lineup over the unmasked lineup, but they developed a preference for the former with experience. Moreover, although prospective confidence ratings were indicative of preference, they were not indicative of identification accuracy (Cutler & Penrod, 1989; Nguyen et al., 2018). Lastly, preference match did not affect identification accuracy—that is, regardless of whether or not a participant preferred the masked lineup, they would, on average, achieve better identification accuracy with the masked lineup over the unmasked lineup.

An additional important finding from Experiment 2 was the replication of the masked lineup superiority for identification when the perpetrator was present. When the perpetrator was absent from the lineup, the two lineup types exhibited comparable correct rejections.

## General discussion

COVID-19 has upended many aspects of daily lives and caused changes to human behaviors at an unprecedented scale. One of the most readily observable changes is the widespread usage of face coverings, which has serious implications for face recognition and eyewitness identification. Despite the extensive literature on both domains, few studies have examined the influence of face coverings on identification (Carlson et al., 2021; Davies & Flin, 1984; Freud et al., 2020; Mansour et al., 2012; Righi et al., 2012), and even fewer were designed to *address* this challenge (Manley et al., 2019). Consequently, there are currently no evidence-based recommendations for how to administer a lineup for crimes involving a masked perpetrator. In the current study, we tested the efficacy of a theoretically-driven, masked lineup procedure. In two experiments, we examined identification accuracy and meta-memory of identifications made from a masked lineup compared to an unmasked lineup. Predictions for this study were borne out of face recognition theories (Tanaka & Simonyi, 2016) in conjunction with the principles of transfer-appropriate processing and context reinstatement (Morris et al., 1977; Smith & Vela, 2001; Tulving & Thomson, 1973).

### Transfer appropriate face processing

In both experiments, a masked lineup increased hit rates compared to an unmasked lineup when the target was present, but it did not increase mistaken identifications when the target was absent. As a result, overall discriminability was significantly better for masked lineups. Further, as evidenced by the overlapping CAC curves, participants exhibited similar calibration across the masked and unmasked lineups. These results support the view that matching the perceptual appearance of the lineup members at retrieval with that at encoding – even if the masked lineup provided participants with *fewer* perceptual details than the masked lineup – improved identification performance.

Decades of face recognition research has tested the holistic account of face recognition. In the past, some authors have used the terms “configural” and “holistic” interchangeably (Richler et al., 2009). However, for the current study, the two terms are separated by the distinction offered by Mauer et al. (2002), such that holistic processing refers to the binding of features into a perceptual Gestalt, whereas configural processing refers to the measurements of spatial relations within and between features. Of particular relevance to the present study is the critique offered by Burton (2013), who argued that a majority of face recognition studies have used computer-generated or hand-drawn faces as stimuli (Manley et al., 2019), which may elicit different perceptual or memory processing than real faces. These static, artificial stimuli also tested what Burton argued as *picture recognition* rather than face recognition. This is a critically important distinction because effective face recognition must happen under variable contexts (Bindemann & Hole, 2020). In the current study, we used a mock crime video at encoding and new photographs in the lineups, thereby providing the type of variable contexts that are commonplace in both everyday face recognition and eyewitness identification situations.

In combination with transfer-appropriate processing, the holistic account of face recognition provides a viable explanation for our results. Note, however, that the purpose of this study was not to test the holistic account per se (i.e., whether faces are processed holistically). Rather, we tested a predicted *application* of the account, according to which a partially-concealed, masked face is not processed holistically. The mask thus forces featural processing of the exposed features (the eyes) during encoding (Freud et al., 2020). This featural processing was then reinstated when a masked lineup was administered, but not when an unmasked lineup—which would prompt holistic processing—was administered.

Our reasoning is that holistic processing at retrieval triggered whole face interference (Leder & Carbon, 2005), which reduced identification accuracy in unmasked lineups compared to masked lineups. This account nicely accommodates the target-present lineup data. It also offers an explanation for why the unmasked lineup did not improve the correct rejection rate when the perpetrator was absent. Specifically, we believe that reinstatement of featural processing during retrieval facilitates participants’ ability to *locate a match* between their memory and the lineup members. In a target-absent lineup, no such matches exist, which would explain the null effect in this condition. To be clear, this explanation is post-hoc, so further research needs to be conducted to test this account. From an application perspective, however, the null effect for target-absent lineups is positive because it suggests that there is little to no drawback to administering a masked face lineup when the target is absent, but there is much to be gained when the target is present.

### Confidence data

With respect to the confidence data, an interesting and somewhat unexpected finding was that the masked lineup increased confidence for both perpetrator identifications and filler identifications, but not for misses or correct rejections. These results differ from those in Manley et al. (2019) and more research is needed to verify its generality and to uncover its explanations. Although the masked lineup increased confidence for identification decisions, it did not affect the diagnostic value of confidence, as evidenced by the overlapping CAC curves for both the masked and unmasked lineups. This is an important finding because confidence is highly persuasive to triers of fact (Cutler et al., 1990; Wells, 1993; Wixted & Wells, 2017).

In addition to examining retrospective confidence, we assessed participants’ meta-memory both before and after they made their identification judgments in Experiment 2. The most striking finding was that unlike retrospective confidence, *prospective confidence* was not at all predictive of accuracy. Indeed, suspect ID accuracy was practically the same regardless of whether participants provided a low (0–40) or high (70–100) prospective confidence rating. Flat CAC curves are rare in eyewitness memory studies, except when participants’ memories have been contaminated by misleading information (Chan et al., 2021), a situation that does not apply here. In fact, participants were asked to make their prospective confidence rating just a few minutes after having seen the crime video, a condition that would qualify for the type of “pristine conditions” that are conducive to producing confidence judgments with high diagnosticity (Wixted & Wells, 2017).

From a theoretical perspective, the dissociation between the prospective and retrospective CAC data suggest that participants used different information to make these confidence judgments. Specifically, we believe that participants made prospective judgments based on a heuristic-based, abbreviated retrieval of the encoding event and the perpetrator’s appearance. This type of retrieval is widely believed to be the basis of delayed judgments of learning and is typically sufficient to produce prospective judgments of high accuracy for relatively simple materials such as paired associates (Nelson & Dunlosky, 1991; Rhodes & Tauber, 2011; Son & Metcalfe, 2005), but it might be inadequate when dealing with the unique challenges associated with eyewitness identification. An important point to consider is the difference between retrieving the identity of a simple item (e.g., the target in a paired associate) and retrieving the perceptual appearance of an unfamiliar face. When studying simple materials such as word pairs, being able to recall the identity of a target item ensures retrieval success (and high confidence). However, recalling the visual details of an unfamiliar face is more difficult, and assessing the quality of the recalled image is far more complex. Although mental images can seem vivid, in reality, their level of details pale in comparison to those of perceived objects (Bainbridge et al., 2021), but these details are precisely the type of information required to distinguish different faces, especially when the task requires one to select amongst faces that all fit a general verbal description (i.e., the hallmark of a fair lineup). Indeed, the fact that we observed such poor diagnosticity for the prospective judgments in a four-trial experiment shows that being familiarized with the eyewitness identification task alone was not enough to improve the diagnostic value of prospective judgments.

Unlike prospective judgments, which are based on a recall heuristic, we believe that retrospective judgments are based on an external recognition experience, such that they are informed by the events that occurred during the identification trial, including how long it took the participant to make a decision, how similar were the lineup members relative to each other and to the participant’s recollection of the target, etc. These tangible experiences provide feedback to participants about their recognition decision and can serve as relatively reliable cues about the accuracy of that decision—something that is not available when one makes a prospective judgment. The information afforded by this experience is not available to individuals when they make prospective judgments, and the poor diagnosticity of the prospective confidence judgments is consistent with other reports (Clark & Tunnicliff, 2001; Cutler & Penrod, 1989; Nguyen et al., 2018; Olsson & Juslin, 1999; Perfect, 2004; Sommer et al., 1995). In sum, prospective confidence appears to be an unreliable indicator of future identification accuracy regardless of whether eyewitnesses are dealing with the more familiar unmasked lineup or the more novel masked lineup; therefore, we argue that prospective confidence judgments should not be used to determine whether or not to administer a lineup.

### Limitations and unknowns

Although administering a masked-face lineup is better than administering a full-face lineup for a masked perpetrator in a laboratory setting, more research needs to be done prior to making a policy recommendation. For instance, participants in this study had a good view of the masked perpetrator. The perpetrator’s face was well-lit, the video was in high definition, and the view of the perpetrator’s face was close to the camera and could be seen from multiple angles. Further, the time between viewing the mock crime and when the lineup was administered was very short (i.e., 5 min). In an investigative setting, the interval between a witnessed event and an identification can range from minutes to years (Semmler et al., 2018), and longer retention will almost certainly reduce correct identification rates, although it is unclear whether it would affect the *pattern* of our results.

Another potential limitation of the current study is that we used only one type of mask. Although a ski mask is a popular method of disguise, there are other types of concealment used by perpetrators (e.g., stocking mask, sunglasses and hats, full-coverage masks, etc.), not to mention the popularity of surgical masks due to COVID-19. Depending on the degree of occlusion and/or distortion of the facial features, the masked-lineup superiority effect is likely to vary. For instance, masks that cover fewer regions of the head (e.g., hair style and forehead are exposed with surgical masks) might produce a masked-lineup superiority effect that is smaller than masks that cover more regions (e.g., one might wear a surgical mask in combination with a hat, which would expose part of the forehead and hair style). Moreover, it is unclear whether sequential exposures to both the masked and unmasked versions of a face would alter the effect (e.g., the lineup administrator might present the unmasked lineup and then ask lineup members to don a mask). In sum, many relevant variables remain unexplored regarding this effect.

### Practical implications

Both of our experiments showed a masked lineup superiority when the perpetrator was in the lineup. This increase in target identification has important implications in a judicial context, given that an eyewitness identification is one of the strongest forms of evidence (Wells, 1993). Manley et al. (2019) was the first study that attempted to address the issue of a identifying a masked perpetrator from a system variable perspective (i.e., a controllable change can be made within the criminal justice system) by testing a novel lineup procedure—albeit with materials that were unrealistic. The current study is the first demonstration of the masked lineup superiority effect in an ecologically realistic design.

Prior to COVID-19, head and face coverings were common only amongst people from Muslim cultures (i.e., a Hijab, Niqab, or a Burqa) and in countries in which mask wearing was a widespread practice to prevent the spread of respiratory illnesses (e.g., Japan). In the context of criminal justice, face masks were worn only by people committing premeditated crimes. But with face coverings having become an essential and global practice, far more unfamiliar face identifications will occur under situations in which a person has previously seen a masked face (e.g., when law enforcement releases photos for persons of interest).

Given that eyewitness misidentification contributes heavily to wrongful convictions, it is important to examine methodologies that may increase identification accuracy. The ultimate goal of eyewitness identification research is to encourage investigative approaches that improve the probative value of eyewitness evidence. One way to do this is to leverage cognitive theories and principles to inform better lineup construction strategies. In the present study, we showed that when a witness encounters a masked perpetrator, administering a masked lineup increases identification accuracy compared to an unmasked lineup. Moreover, with experience, the majority of participants actually prefer a masked lineup. The idea of matching the perceptual appearance of lineup members to the perceptual appearance of the originally encoded face when disguises were involved was vaguely considered decades ago (Cutler, 1988). With the advances in eyewitness identification research, this idea warrants more serious consideration—as it could prove a powerful way to improve the accuracy of everyday face recognition in general and eyewitness identification in particular.

## Data Availability

The dataset supporting the conclusions of this article is available on the first author’s OSF page (https://osf.io/5f68j/?view_only=912a09bd44954de58c41336a7b74fa33).

## Footnotes

1 It is unclear whether and to what extent holistic processing is invoked when a person views a partial face, or how much of a face needs to be visible to elicit holistic processing (McKone et al., 2006). What is clear is that misaligning, removing, or occluding features of a face can disrupt holistic processing (Freud et al., 2020; Moscovitch et al., 1997). One might note that Fitousi et al. (2021) reached the opposite conclusion as McKone et al., with Fitousi et al. arguing that masking does not disrupt holistic processing. Our examination of their data, however, suggests that such a conclusion might be unwarranted because (1) the results of Fitousi et al. are similar to those reported by McKone et al., and (2) Fitousi et al. had about a quarter of the sample size relative to McKone et al. Even if participants had processed a masked face holistically in our study, that representation would not be reinstated at retrieval when the face became unmasked. Therefore, our prediction does not change regardless of whether one subscribes to the view of holistic processing, configural processing, third-order relations, etc., because the exact nature of face processing with or without masks is not the focus our study. Rather, we are interested in the influence of matching/mismatching during encoding and retrieval for face identification.
